# Advances in Metal-Catalyzed Cross-Coupling Reactions of Halogenated Quinazolinones and Their Quinazoline Derivatives

**DOI:** 10.3390/molecules191117435

**Published:** 2014-10-29

**Authors:** Malose Jack Mphahlele, Marole Maria Maluleka

**Affiliations:** Department of Chemistry, College of Science, Engineering and Technology, University of South Africa, P.O. Box 392, Pretoria 0003, South Africa; E-Mail: malulmm@unisa.ac.za

**Keywords:** halogenoquinazolin-4-ones, halogenoquinazolines, cross-coupling reactions

## Abstract

Halogenated quinazolinones and quinazolines are versatile synthetic intermediates for the metal-catalyzed carbon–carbon bond formation reactions such as the Kumada, Stille, Negishi, Sonogashira, Suzuki-Miyaura and Heck cross-coupling reactions or carbon-heteroatom bond formation via the Buchwald-Hartwig cross-coupling to yield novel polysubstituted derivatives. This review presents an overview of the application of these methods on halogenated quinazolin-4-ones and their quinazolines to generate novel polysubstituted derivatives.

## 1. Introduction and Scope

**Figure 1:**
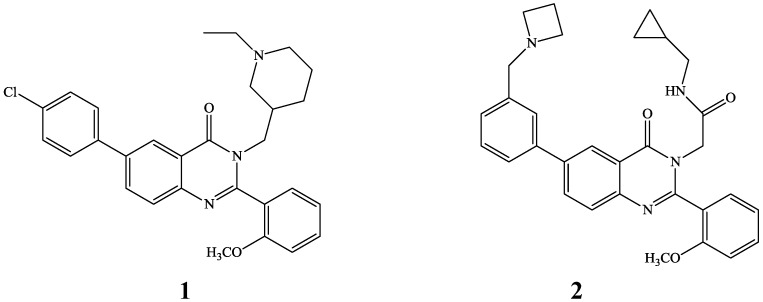
Examples of biologically-relevant polycarbo-substituted quinazolin-4(3*H*)-ones.

In recent years, the development of strategies to efficiently functionalize presynthesized halogenated quinazolinones and quinazolines or their tosylate derivatives via metal-catalyzed cross-coupling reactions to afford novel polycarbo- or polyheteroatom-substituted derivatives with potential application in pharmaceuticals and materials has attracted considerable interest. The 3-substituted 2,6-diarylquinazolin-4(3*H*)-ones **1** and **2** (Figure 1), for example, were previously prepared via Suzuki-Miyaura cross-coupling of the corresponding 6-halogenated 4(3*H*)-oxo precursors and were found to be ghrelin receptor and vasopressin V_1b_ receptor antagonists, respectively [1,2].

**Figure 2:**
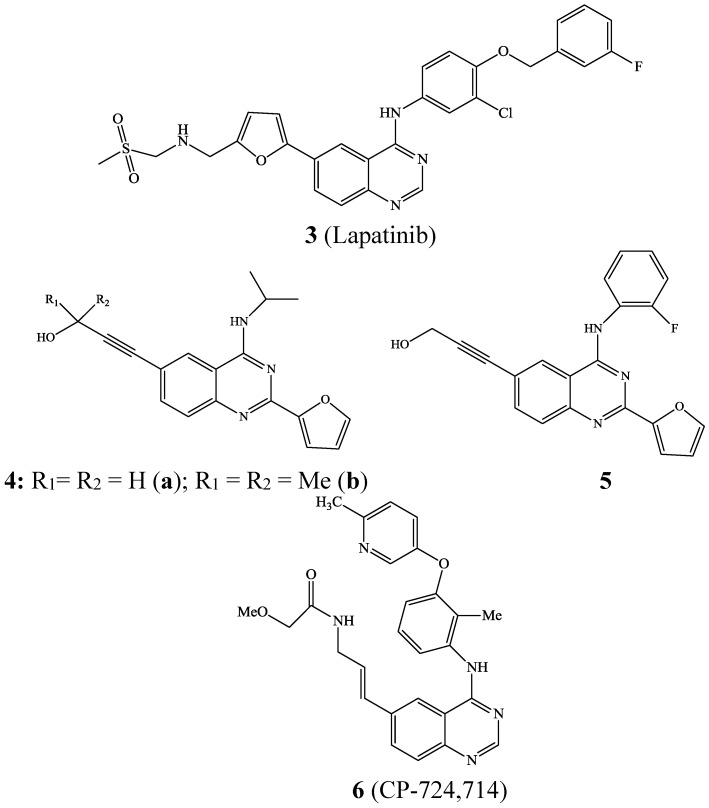
Carbo-substituted 4-(aryl/alkylamino)quinazolines of biological importance.

Lapatinib **3**, a 6-heteroaryl substituted 4-anilinoquinazoline derivative is an oral dual tyrosine kinase inhibitor (TKI) that targets both EGFR and HER2 to inhibit the proliferation of breast cancer cells [3]. The 6-alkynylated 4-aminoquinazolines **4** and **5** (Figure 2), on the other hand, serve as selective inhibitors of Aurora A [4]. Likewise, the 4-anilinoquinazoline derivative **6** (CP-724,714) is a selective ErbB2 angiogenesis inhibitor under investigation for the treatment of breast, ovarian and other types of cancer [5].

**Figure 3:**
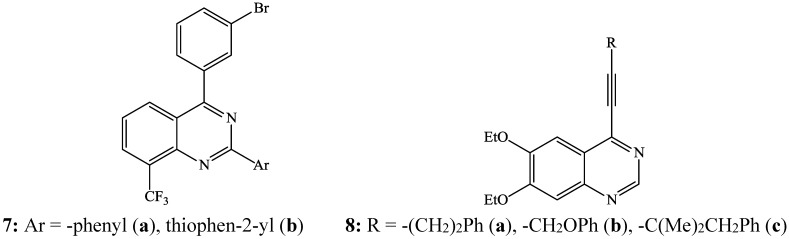
Examples of biologically active 4-aryl- **7** and 4-alkynylquinazolines **8**.

During the course of the exploration of non-anilinoquinazoline scaffold, trisubstituted quinazoline derivatives such as 4-(3-bromophenyl)-8-(trifluoromethyl)-2-phenylquinazoline **7a** and 4-(3-bromophenyl)-2-(thiophen-2-yl)-8-(trifluoromethyl)quinazoline **7b** (Figure 3) were prepared as part of a series of liver X-receptor modulators [6]. Likewise, the 4-alkynylquinazolines **8** were found to be potent epidermal growth factor receptor (EGFR) tyrosine kinase inhibitors [7].

New findings on the biological and photophysical properties of polycarbo-substituted quinazolinones and quinazolines reveal a need to increase the diversity of substituents around these heterocyclic scaffolds. To this end, halogenated quinazolinones and quinazoline derivatives have established themselves as important intermediates for the synthesis of polycarbo- and polyheteroatom-substituted derivatives with potential application in material and medicinal chemistry. The versatility of the halogenated heterocycles as synthetic intermediates is a consequence of C*sp*^2^–X bonds, which enable further elaboration into polycarbo-substituted or polyheteroatom-substituted derivatives. Despite the importance of metal catalysed cross-coupling reactions in synthesis, there is no comprehensive review in the literature on the application of these methods on halogenated quinazolinones and quinazolines to afford polysubstituted derivatives. Only a few examples of transition metal mediated cross-coupling of halogenated quinazolinones or quinazolines are described in a monograph by de Vries [8] and the review paper by Connolly *et al.* [9]. Most of the review articles in the literature on quinazolinones and quinazolines, on the other hand, only focus on the conventional and microwave syntheses of heteroatom-substituted derivatives as well as their medicinal applications [10,11,12]. This prompted us to compile a comprehensive review on the application of the Kumada, Stille, Negishi, Sonogashira, Suzuki-Miyaura and Heck cross-coupling as well as the Buchwald-Hartwig cross-coupling and palladium-catalysed cyanation in the synthesis of polycarbo- or polyheteroatom-substituted quinazolinones and quinazolines derivatives from the corresponding halogenated precursors.

## 2. Reactivity of Halogenated Quinazolinones and Quinazoline Derivatives in Cross-Coupling Reactions

The use of halogenated quinazolinones and quinazolines as intermediates in transition metal-catalyzed cross-coupling reactions to form C*sp*^2^–C*sp*^2^, C*sp*^2^–C*sp* or C*sp*^2^–heteroatom bond(s) takes advantage of the ease of displacement of iodine, bromine, or chlorine atom(s) on the aryl or heteroaryl moiety by nucleophiles or metal catalysts. The order of reactivity of C*sp*^2^–halogen bonds in transitional metal-mediated cross-coupling for aryl/heteroaryl halides, C-I > C-Br >> C-Cl, generally allows selective coupling with iodides or bromides in the presence of chlorides [13,14]. Theoretical calculations at B3LYP level, on the other hand, reveal that the bond dissociation energy of the C(4)-Cl bond (84.8 kcal/mol) of 6-bromo-2,4-dichloroquinazoline is larger than that of the weaker C*sp*^2^-Br bond (83 kcal/mol at B3LYP) [15]. The C*sp*^2^–Cl bond of the 4-chloroquinazoline moiety is, however, highly activated relative to other chlorinated or brominated positions due to α-nitrogen effect and additional activation resulting from the coordination of palladium(0) with the N-3 lone pair electron density in the oxidative-addition step [15,16,17]. Among the cross-coupling reactions with organometallic reagents that involve halogenoquinazolinones and halogenoquinazolines as well as their tosylate derivatives, Sonogashira and Suzuki-Miyaura cross-coupling reactions and to some extent the Heck reaction are more prevalent. Only limited examples involving the application of the Kumada, Stille and Negishi cross-coupling reactions in the synthesis of polycarbo-substituted quinazolinones and quinazolines exist in the literature. This also applies for the miscellaneous methods such as Buchwald-Hartwig cross-coupling and palladium catalysed cyanation of halogenated quinazolinones and quinazoline derivatives.

## 3. Application of Cross-Coupling Reactions in the Synthesis of Substituted Quinazolinones and Quinazolines

The development of metal-catalyzed cross-coupling reactions over the past decade has revolutionized the way carbon-carbon and carbon-heteroatom bonds are formed. Kumada, Negishi, Heck, Suzuki-Miyaura, Stille and Sonogashira cross-coupling reactions represent powerful synthetic tools for the construction of carbosubstituted quinazolinones and/or quinazolines. The Buchwald-Hartwig cross-coupling and Pd-catalyzed cyanation, on the other hand, have also been employed for the synthesis of novel heteroatom- or cyano-substituted quinazolinones and quinazoline derivatives.

### 3.1. Application of Kumada Cross-Coupling Reaction in the Synthesis of Polysubstituted Quinazolines

**Scheme 1:**
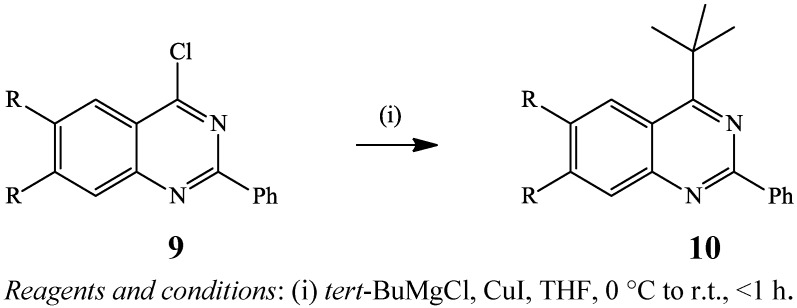
CuI-catalyzed cross-coupling of **9** with *tert-*butylmagnesium chloride.

Kumada cross-coupling involves the reaction of arylhalides or triflates with Grignard reagents in the presence of palladium catalyst. Despite its relatively early discovery, the application of the Kumada reaction is only limited to the transformation of halogenated quinazolines because of the incompatibility of Grignard reagents with the amide group of quinazolinones. The reducing ability of the Grignard reagents, on the other hand, has been found to cause the precipitation of palladium black and, in turn, arrest the catalytic turnover. A modification of this reaction involving the cross-coupling of 2,4-dichloroquinazoline (R = H) or its 2,4-dichloro-6,7-dimethoxyquinazoline derivative (R = OMe) **9** with *tert*-butylmagnesium chloride in the presence of copper(I) iodide as catalyst in tetrahydrofuran (THF) at room temperature afforded the 4-substituted quinazoline derivatives **10a** (92%) and **10b** (63%) in less than an hour (Scheme 1) [18]. The selectivity of C*sp*^2^–C*sp*^2^ bond formation in this case is attributed to the increased reactivity of the C-4 position of the quinazoline ring due to the α-nitrogen effect. Anhydrous manganese chloride (MnCl_2_) or manganese chloride tetrahydrate (MnCl_2_.4H_2_O) have also been employed to promote the cross-coupling of 4-chloro-2-phenylquinazoline with phenylmagnesium chloride in THF for 1.5 h to afford 2,4-diphenylquinazoline in 71% yield [19]. Despite its efficiency, this method makes use of an excess of the Grignard reagent (4 equiv.), which only tolerates limited functional groups on both of the coupling partners. The mild reaction conditions as well as the lower cost of copper(I) iodide and manganese chloride nevertheless, render these modified methods an attractive alternative for the synthesis of carbosubstituted quinazolines using palladium or nickel salts.

*Reagents and conditions*: (i) *tert*-BuMgCl, CuI, THF, 0 °C to r.t., <1 h.

The high nucleophilicity of the readily accessible Grignard reagents generally limits the application of the Kumada cross-coupling towards the synthesis of novel polycarbo-substituted quinazolinones.

### 3.2. Application of Negishi Cross-Coupling in the Synthesis of Polysubstituted Quinazolines

**Scheme 2:**
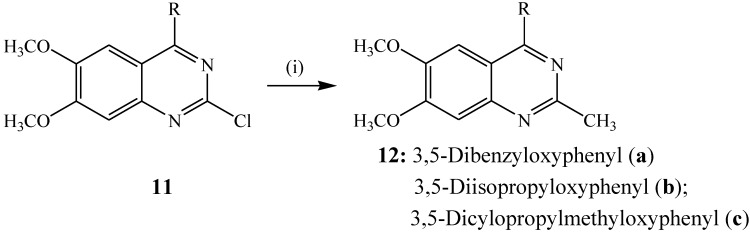
Negishi cross-coupling of **11** with MeZnCl.

The Negishi cross-coupling reaction of aryl or vinyl halides/triflates with organozinc reagents in the presence of palladium [Pd^0^, Pd^2+^] or nickel source [Ni^0^, Ni^2+^] as catalyst and a phosphine ligand is a versatile and efficient method for the synthesis of a variety of heterocyclic motifs [20]. Organozinc reagents can be prepared either from the corresponding organohalide (RX) by reductive metalation [21] or from other organometalic compounds, often RLi, by transmetalation [22]. The cross-coupling reaction normally occurs at or slightly above room temperature to avoid the degradation of the zinc compound at high temperature. This reaction has thus far only been employed for the synthesis of carbo-substituted quinazolines from the corresponding halogenated precursors. The Negishi cross-coupling between the 4-substituted 2-chloro-6,7-dimethoxyquinazolines **11** and CH_3_ZnCl reagent generated *in situ* from methyl lithium and zinc(II) chloride in the presence of tetrakis(triphenylphosphine)palladium(0) (Pd(PPh_3_)_4_) as a Pd(0) catalyst source in dioxane, for example, previously afforded compounds **12** (Scheme 2) [17].

*Reagents and conditions*: (i) MeLi, ZnCl_2_, Pd(PPh_3_)_4_, dioxane.

**Scheme 3:**
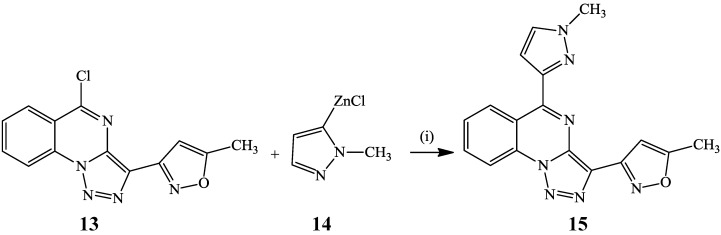
Negishi cross-coupling of **13** with **14**.

Negishi cross-coupling of **13** with 5-(1*-N*-methylpyrazole)zinc chloride **14** under palladium catalysis afforded triazoloquinazoline **15** in 40% yield (Scheme 3) [23].

**Scheme 4:**
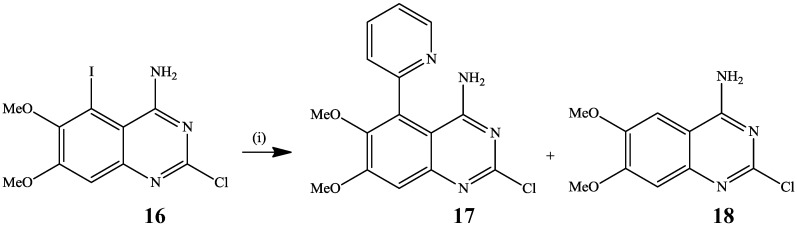
Negishi cross-coupling of 2-chloro-5-iodo-6,7-dimethoxyquinazolin-4-amine **16**.

*Reagents and conditions*: (i) Pd(PPh_3_)_4_, DMF, 50 °C.

(2-Pyridyl)zinc chloride prepared, in turn, from 2-bromopyridine by halogen-metal exchange with isopropylmagnesium chloride followed by addition of zinc chloride [24], was reacted with 2-chloro-5-iodo-6,7-dimethoxyquinazolin-4-amine **16** in the presence of palladium acetate-triphenylphosphine catalyst complex in THF under reflux to afford a mixture of the cross-coupled product **17** and the reduced derivative **18** (Scheme 4) [25]. The latter was formed as a sole product by treatment of **16** with 2-iodopyridine in the presence of activated zinc and Pd(OAc)_2_–PPh_3_ mixture in DMF.

*Reagents and conditions*: (i) 2-PyZnCl, Pd(OAC)_2_, PPh_3_, THF, reflux.

Despite high sensitivity of organozinc reagents to air and moisture, their high compatibility with various functional groups make the Negishi cross-coupling reaction a powerful tool for the formation of carbon-carbon bonds.

### 3.3. Application of Stille Cross-Coupling in the Synthesis of Polysubstituted Quinazolines

**Scheme 5:**
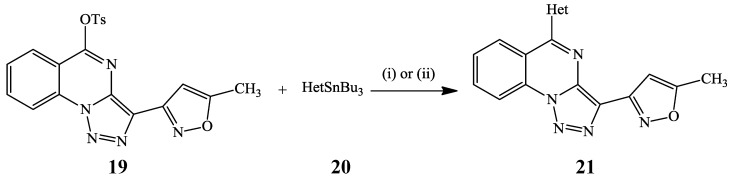
Stille cross-coupling of 5-tosyltriazoloquinazoline **19** with heterarylstannanes.

The Stille cross-coupling which involves the reaction between organostannanes and organic halides or tosylates in the presence of Pd catalyst [26] has thus far only been employed for the synthesis of polysubstituted quinazolines from the corresponding halogenated precursors. 5-Chlorotriazoloquinazoline, for example, was subjected to Stille cross-coupling with heterarylstannanes using Pd(PPh_3_)_4_-CuI catalyst mixture in DMF at 70 °C to afford a series of 5-heteraryl-substituted triazoloquinazolines in 20%–78% yields [23]. Under the same reaction conditions, the analogous 5-tosyltriazoloquinazoline **19** reacted with heterarylstannanes **20** to afford the corresponding 5-heteraryl–substituted derivatives **21** in reasonable yields (Scheme 5) [23].

*Reagents and conditions*: (i) Pd(PPh_3_)_4_, DMF, 70 °C, 8 h; (ii) Pd(PPh_3_)_4_, CuI, DMF, 70 °C, 1 h.

**Table:** 

	Het	%Yield Condition (i)	%Yield Condition (ii)
**21a**	2-thienyl	91	78
**21b**	2-furyl	73	87
**21c**	2-(*N*-methylimidazole)	-	52
**21d**	5-(*N*-methyl-[1,2,4]-triazole)	-	69

**Scheme 6:**
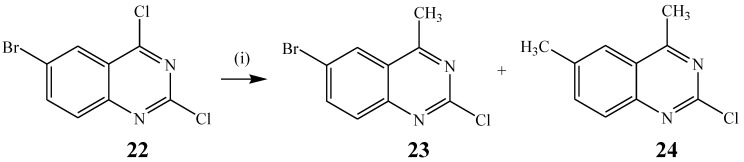
Regioselective alkylation of 6-bromo-2,4-dichloroquinazoline **22**.

A modification of the Stille cross-coupling involving the use of 6-bromo-2,4-dichloroquinazoline **22** as substrate and trimethylalane (1.2 equiv.) as coupling partner in the presence of Pd(PPh_3_)_4_ as a source of active Pd(0) catalyst to afford a mixture of the C-4 substituted **23** (47%) and C-6 cross-coupled product **24** (16%) has been described before (Scheme 6) [16]. The preponderance of the C-4 cross-coupled product is a consequence of the increased reactivity of the C(4)–Cl bond due to the α-effect and strong coordination of Pd(0) with N-3 lone pair electrons in the oxidative-addition step. The 2-position of the pyrimidine ring, on the other hand, is generally known to be less reactive to oxidative addition of Pd than the 4-position [27].

*Reagents and Conditions*: (i) Al(CH_3_)_3_, Pd(PPh_3_)_4,_ THF, reflux.

### 3.4. Application of Sonogashira Cross-Coupling in the Synthesis of Polysubstituted Quinazolinones and Quinzoline Derivatives

**Scheme 7:**
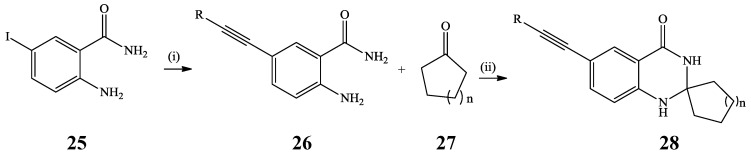
Sonogashira cross-coupling of **25** and subsequent cyclocondensation of **26** and **27**.

The Sonogashira cross-coupling of aryl halides or tosylates with terminal acetylenes in the presence of Pd(0) catalyst source and copper(I) iodide (CuI) as co-catalyst provides an effective route for C*sp*^2^-C*sp* bond formation to afford arylalkynes and conjugated enynes [28]. A combination of palladium and copper as catalysts results in the increased reactivity of the reagents and as a result, Sonogashira cross-coupling can also be carried out at room temperature [28]. This reaction has been employed extensively in the synthesis of alkynylated quinazolinones and quinazolines with potential application in medicinal and material chemistry. Initial Pd/C-PPh_3_-CuI catalyzed Sonogashira cross-coupling of 2-amino-5-iodobenzamide **25** with terminal acetylenes in the presence of triethylamine as a base in ethanol under reflux afforded the 5-alkynyl-2-aminobenzamides **26** (Scheme 7) [29]. The latter were, in turn, subjected to cyclocondensation with cycloalkanones **27** using Amberlyst-15 as a catalyst in acetonitrile under ultrasonic irradiation at room temperature for 2–3 min. to afford a series of spiro 6-alkynyl-2,3-dihydroquinazolin-4(1*H*)-ones **28**.

*Reagents and conditions*: (i) R-C≡CH, 10% Pd/C, PPh_3_, CuI, NEt_3_, EtOH, 70 °C, 4 h; (ii) Amberlyst-15, ultrasound irradiation (40 KHz), CH_3_CN, r.t., 2–3 min.

**Table:** 

	n	R	%Yield
**28a**	1	Ph	98
**28b**	2	Ph	98
**28c**	3	Ph	95
**28d**	2	-CH_2_CH_2_I	97

**Scheme 8:**
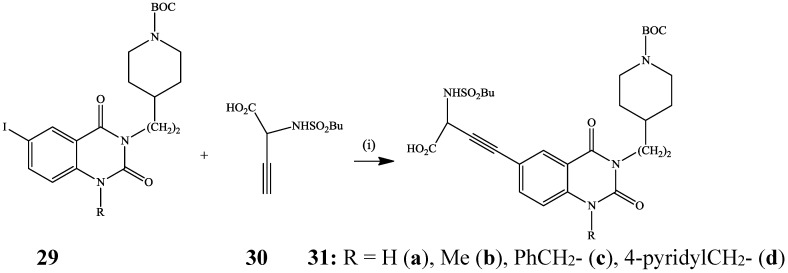
Sonogashira cross-coupling of **29** with **30**.

The Sonogashira cross-coupling of 6-iodoquinazolinedione **29** or its NCH_3_-4(3*H*)-oxo derivative with ethyl 2-(1-butanesulfonamido)pent-4-yn-l-oate **30** in the presence of Pd(PPh_3_)_4_-CuI catalyst mixture previously afforded the cross-coupled products **31** as potent fibrinogen receptor antagonists (Scheme 8) [30].

*Reagents and conditions*: (i) Pd(PPh_3_)_4_, Cul, HNEt_2_, 40 °C.

**Scheme 9:**
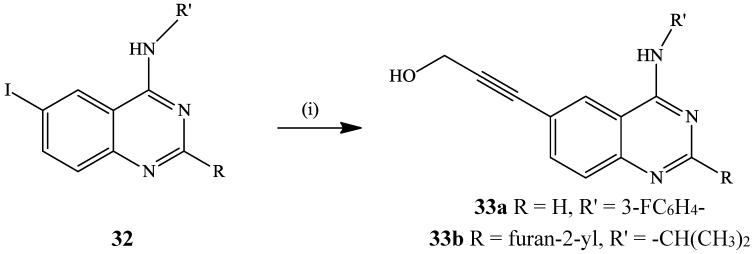
Sonogashira cross-coupling of **32** with propargyl alcohol.

Sardon *et al.* subjected the 6-iodo-*N*-isopropylquinazolin-4-amines **32** to Sonogashira cross-coupling with propargyl alcohol in the presence of PdCl_2_(PPh_3_)_2_-1,1-bis(diphenylphosphino)ferrocene catalyst complex, CuI as co-catalyst and NEt_3_ in DMF at 50 °C to afford the corresponding 6-alkynylated quinazoline-4-amines **33a** and **33b** with selective inhibition of AurA *vs.* AurB (Scheme 9) [4]. Under the same reaction conditions, *N*-(3-fluorophenyl)-6-iodoquinazolin-4-amine also coupled with 2-methylbut-3-en-2-ol to yield the 6-alkynylated derivative in 73% yield [4].

*Reagents and conditions*: (i) PdCl_2_(PPh_3_)_2_, 1,1-bis(diphenylphosphino)ferrocene, CuI, NEt_3_, DMF, 50 °C.

**Scheme 10:**
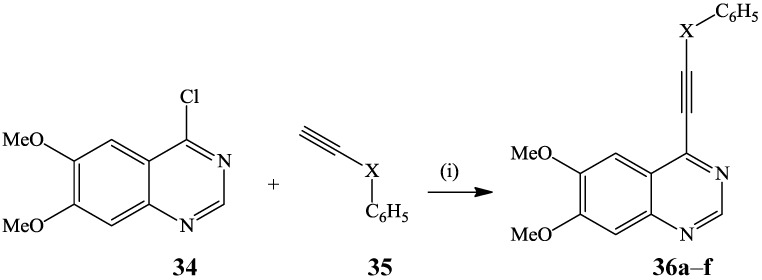
Sonogashira cross-coupling of 6,7-disubtituted-4-chloroquinazoline **34**.

Alkynylation of 4-chloro-6,7-dimethoxyquinazoline **34** with various terminal alkynes **35** (1.5 equiv) in the presence of palladium catalyst, CuI as a co-catalyst and NEt_3_ as a base in DMF afforded the 4-alkynylquinazolines **36** as scaffolds for potent epidermal growth factor receptor (EGFR) tyrosine kinase inhibitors (Scheme 10) [7].

*Reagents and conditions*: (i) Pd(PPh_3_)_4_ or PdCl_2_(PPh_3_)_2_, CuI, NEt_3_, DMF, r.t.

**Table:** 

	X	EGFR TK, IC_50_ (nM)
**36a**	0	5600
**36b**	-(CH_2_)_2_-	14
**36c**	-CH_2_O-	15
**36d**	-C(Me_2_)CH_2_-	13
**36e**	-C(Me_2_)N(Me)-	80
**36f**	-(CH_2_)_3_-	3

**Scheme 11:**
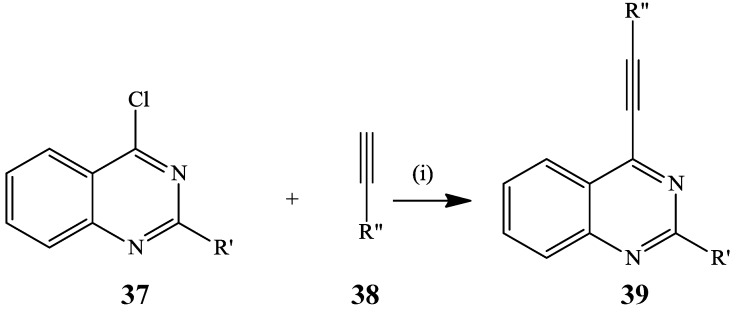
Sonogashira cross-coupling of 37.

Sonogashira cross-coupling of the 2-substituted 4-chloroquinazolines **37** with terminal alkynes **38** using Pd(PPh_3_)_4_ and CuI in the presence of cesium carbonate (Cs_2_CO_3_) in dry DMF at room temperature afforded a series of the corresponding 4-alkynylquinazolines **39** in high yields (Scheme 11) [31].

*Reagents and conditions*: (i) Pd(PPh_4_)_3_, CuI, Cs_2_CO_3_, DMF, r.t., 3 h.

**Table:** 

	R'	R''	%Yield
**39a**	-CH_3_	Ph	90
**39b**	-CH_3_	Cyclopropyl	98
**39c**	H	Ph	95
**39d**	-CF_3_	Ph	75
**39e**	-CF_3_	Cyclopropyl	60
**39f**	H	Cyclopropyl	72

**Scheme 12:**
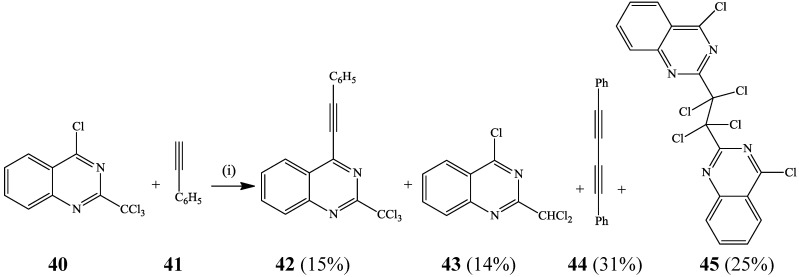
Sonogashira cross-coupling of 4-chloro-2-trichloromethylquinazoline **40**.

Attempted Sonogashira cross-coupling of 4-chloro-2-trichloromethylquinazoline **40** with phenylacetylene **41** using triethylamine as a base, Pd(PPh_3_)_4_ as a source of the reactive Pd(0) species and CuI as a co-catalyst in THF did not afford the expected cross-coupled product [31]. The use of Cs_2_CO_3_ as a base, Pd(OAc)_2_ as a catalyst in DMF, on the other hand, afforded the cross-coupled product **42** in low yield (15%) along with other undesirable products **43**–**45** (Scheme 12). The presence of trichloromethyl group at position 2 of **40** was found to complicate the outcome of this reaction. Likewise, 4-bromo- and 4-iodo-2-trichloromethylquinazolines prepared, in turn, from 2-trichloromethyl quinazolin-4(3*H*)-ones using tetrabutylammonium bromide (TBABr) and tetrabutylammonium iodide (TBAI) in the presence of P_2_O_5_ in toluene under reflux were cross-coupled with cyclopropylacetylene and phenylacetylene to afford the corresponding 4-alkynyl-2-trichloromethylquinazolines in 15% and 9%, respectively [31]. These poor yields indicate that the 4-chloroquinazoline moiety is preferred over the 4-bromo- or 4-iodoquinazoline framework in the cross-coupling reactions.

**Scheme 13:**
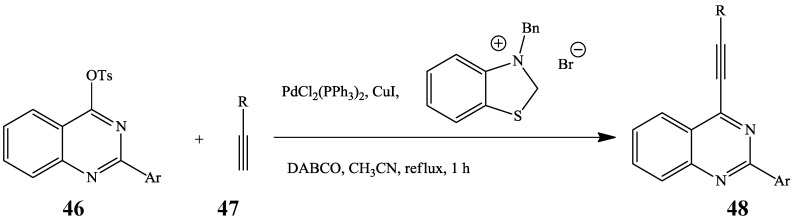
Sonogashira cross-coupling of quinazoline-4-tosylates **46**.

6-Tosyl-[1,2,3]-triazoloquinazoline derivative has been cross-coupled with trimethylsilylacetylene in the presence of PdCl_2_(PPh_3_)_2_ and CuI in DMF at 60 °C to afford the 5-trimethylsilanylethynyl-[1,2,3]-triazoloquinazoline in 88% yield [23]. Sonogashira cross-coupling of the analogous 2-arylquinazoline-4-tosylates **46** with terminal alkynes **47** (R = aryl, cyclopropyl, *n-*butyl or cyclohexen-1-yl) using *N*-heterocyclic carbenes (NHC) as ligands in the presence of Pd(0)–Cu catalyst complex recently afforded a series of 4-alkynyl-2-arylquinazolines **48** in 58%–95% yields (Scheme 13) [32]. The NHC ligands have been found to exhibit similar electronic properties to phosphines, being strongly σ-donating and weakly π-acidic and also to offer very high catalytic activity combined with stability and longevity in comparison with phosphine ligands [33].

*Reagents and conditions*: (i) Cs_2_CO_3_, Pd(OAc)_2_, CuI, DMF, r.t., 3 h.

**Scheme 14:**
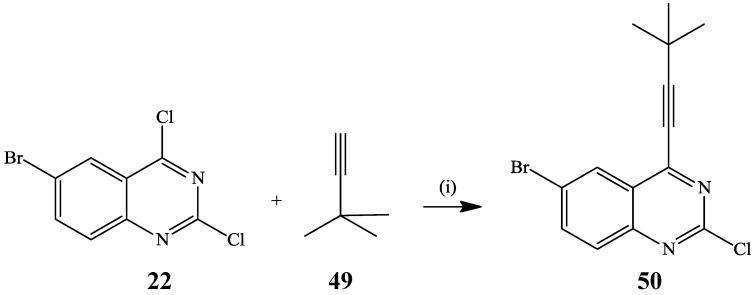
Site-selective C-4 alkynylation of 6-bromo-2,4-dichloroquinazoline **22**.

Sonogashira cross-coupling of 2,4-dichloroquinazoline [15] and 6-bromo-2,4-dichloroquinazoline [34] with stoichiometric amount of terminal alkynes previously led to exclusive replacement of the 4-chloro atom. Selective mono-alkynylation of 6-bromo-2,4-dichloroquinazoline **22** with *tert*-butyl acetylene **49** to afford the C-4 substituted product **50** in 67% yield was achieved in the presence of PdCl_2_(PPh_3_)_2_-CuI catalyst mixture and triethylamine as a base in THF at room temperature (Scheme 14) [17]. Likewise, Sonogashira cross-coupling of 2,4-dichloroquinazoline with 4-dimethylaminophenylacetylene (2.5 equiv.) in the presence of PdCl_2_(PPh_3_)_2_-CuI catalyst mixture in diisopropylamine at 70 °C for 15 h afforded the 4-alkynylated derivative, exclusively [35].

*Reagents and conditions*: (i) NEt_3_, PdCl_2_(PPh_3_)_2_, CuI, THF, r.t., 20 h.

**Scheme 15:**
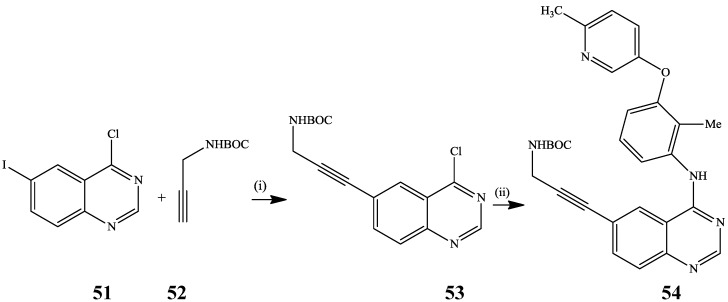
Site-selective alkynylation of **51** and subsequent amination of **53**.

Ripin *et al.*, previously subjected 4-chloro-6-iodoquinazoline **51** to BOC-protected propargylamine **52** in the presence of PdCl_2_(PPh_3_)_2_-CuI catalyst mixture and *i*-Pr_2_NH in THF at r.t. for 3 h followed by amination of the incipient 6-alkynylated-4-chloroquinazoline **53** to afford the alkynylated anilinoquinazoline derivative **54** (Scheme 15) [5]. However, the propargylamine used and the reaction conditions outlined in the scheme caused the target compound to differ from those described in the Experimental Section. This example, nevertheless, represents the first and only reported site-selective alkynylation involving a chloro-iodo quinazoline derivative as a substrate. This is because most of the site selectivity involving di- or trihalogeno quinazolines focus on the reactivity of the chloro-chloro or chloro-bromo substituted derivatives. Sonogashira cross-coupling of 2,4-diamino-4-iodoquinazoline with methyl 4-ethynylbenzoate catalysed by Pd(OAc)_2_-tris(2-tolyl)phosphine-CuI catalyst mixture in the presence of NEt_3_ in DMF at 60 °C for 24 h also afforded the 6-alkynylated derivative in 95% yield [36].

*Reagents and conditions*: (i) PdCl_2_(PPh_3_)_2_, CuI, *i*-Pr_2_NH, THF, r.t., 3 h; (ii) 3-(6-methylpyridin-3-yloxy)-2-methyl benzenamine, *t*-BuOH, dichloroethane, 80 °C, 1 h.

**Scheme 16:**
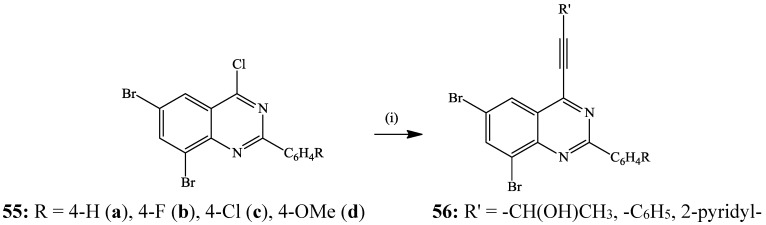
Site-selective Sonogashira cross-coupling of **55**.

*Reagents and conditions*: (i) R'C≡CH, Pd(PPh_3_)_4_, CuI, Cs_2_CO_3_, THF, r.t., 24 h.

The 2-aryl-6,8-dibromo-4-chloroquinazolines **55** derived from the corresponding 2-aryl-6,8-dibromoquinazolin-4(3*H*)-one precursors have recently been found to undergo Sonogashira cross-coupling with terminal acetylenes (propargyl alcohol, phenylacetylene and 2-pyridinylacetylene) through exclusive replacement of the 4-chloro atom to afford the 4-alkynyl-6,7-dibromoquinazolines **56** in 53%–72% yield (Scheme 16) [37]. The increased reactivity of the C(4)-Cl bond observed for the bromo-chloro substituted quinazolines **22** and **55** is attributed to α-nitrogen effect, which makes the C-4 position highly activated than the other positions bearing Cl or Br [15,34].

### 3.5. Application of Heck Cross-Coupling in the Synthesis of Polysubstituted Quinazolinones and Quinazolines

**Scheme 17:**
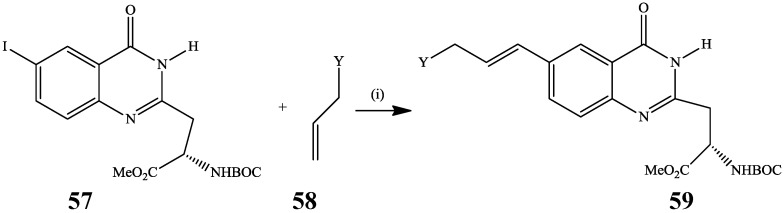
Heck cross-coupling of **57** with allyl amidines or guanidines.

The Heck reaction which involves Pd-catalyzed [Pd(PPh_3_)_4_, PdCl_2_(PPh_3_)_2_, or Pd(OAc)_2_] carbon-carbon bond formation through inter- or intramolecular cross-coupling reaction between organohalides or triflates with alkenes is a powerful tool for the construction of alkenyl- and aryl-substituted alkenes. 2-Substituted 6-iodoquinazolin-4(3*H*)-one **57** was previously reacted with unprotected allyl amidines or guanidines **58** in the presence of palladium acetate as active Pd(0) source, tri-(*o-*tolyl)phosphine as ligand and triethylamine in acetonitrile at 80 °C to afford a series of the 6-vinyl substituted products **59** as potential vitronectin receptor (α_ν_β, integrin) antagonists (Scheme 17) [38]. To our knowledge, this represents the only example of the application of the Heck cross-coupling reactions on halogenated quinazolinones to afford alkenyl-substituted derivatives.

*Reagents and conditions*: (i) Pd(OAc)_2_, (*o-*tolyl)_3_P, NEt_3_, CH_3_CN, 80 °C.

**Table:** 

	Y	%Yield
**59a**	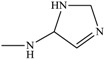	77
**59b**	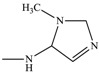	79
**59c**	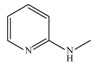	81
**59d**	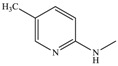	56
**59e**	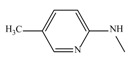	62
**59f**	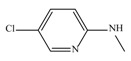	54
**59g**	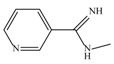	66
**59h**	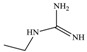	78

**Scheme 18:**
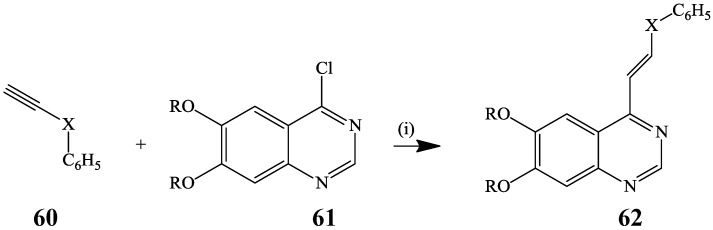
Heck-type cross-coupling of incipient hydro-zirconated **60** with **61**.

The Heck cross-coupling reaction has, however, been applied extensively on the synthesis of poly-carbosubstituted derivatives from the corresponding halogenated quinazoline precursors. For example, initial hydro-zirconation of the terminal alkynes **60** followed by a Heck-type cross-coupling involving the reaction of the incipient alkene-like intermediate with 6,7-dialkoxy-4-iodoquinazolines **61** in THF at room temperature afforded the analogous 4-alkenyl-6,7-dialkoxyquinazolines **62** (Scheme 18) [7]. Of interest is that compounds **62** serve as epidermal growth factor receptor (EGFR) tyrosine kinase inhibitors [7].

**Scheme 19:**
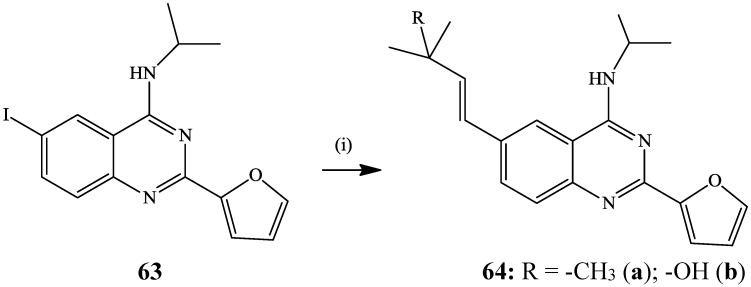
Heck cross-coupling of 6-iodo-4-(isopropylamino)quinazolin-6-yl)acrylates **63**.

The Heck cross-coupling of 2-(furan-2-yl)-6-iodo-*N*-isopropylquinazolin-4-amine **63** with *tert*-butyl acrylate or 2-methylbut-3-en-2-ol in the presence of Pd(OAc)_2_–tri-*o*-tolylphosphine catalyst complex and NEt_3_ in acetonitrile at 100 °C afforded the 6-alkenyl-4-(isopropylamino)quinazolin-6-yl)acrylates **64a** and **64b** in 63% and 86% yield, respectively (Scheme 19) [4]. Under the same reaction conditions, *N*-(3-fluorophenyl)-6-iodoquinazolin-4-amine reacted with 2-methylbut-3-en-2-ol to afford the 6-alkenylated derivative in 73% yield [4].

*Reagents and conditions*: (i) Cp_2_Zr(H)Cl, Pd(PPh_3_)_4_, THF, r.t.

**Table:** 

	R	X	EGFR TK, IC_50_ (nM)
**62a**	-Me	-(CH_2_)_2_-	4
**62b**	-Me	-C(Me)N(Et_2_)	30
**62c**	-Me	0	15
**62d**	-Et	-(CH_2_)_2_-	5
**62^e^**	-Et	-(CH_2_)_3_-	140

*Reagents and conditions*: (i) alkene, NEt_3_, Pd(OAc)_2_, (*o*-Tol)_3_P, acetonitrile, 100 °C, 48–72 h.

**Scheme 20:**
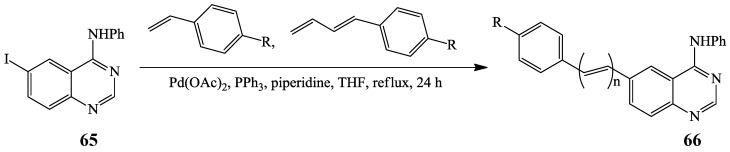
Heck cross-coupling of **65** with styrene or phenylbutadiene.

6-Iodo-*N*-phenylquinazolin-4-amine **65** was recently cross-coupled with styrene or phenylbutadiene derivatives in the presence of Pd(OAc)_2_-PPh_3_ catalyst complex and piperidine in THF under reflux to yield the corresponding 6-carbosubstituted *N*-phenylquinazolin-4-amines **66** albeit in low yields (Scheme 20) [38]. (*E*)-6-(4-Dimethylaminostyryl)-*N-*phenylquinazolin-4-amine **66a** was found to exhibit ERBB2-induced fluorescence thus demonstrating its utility as a turn-on fluorescence kinase inhibitor in MCF7 cells [39].

**Table:** 

	n	R	% Yield
**66a**	1	-N(CH_3_)_2_	16
**66b**	1	-OMe	25
**66c**	1	-H	16
**66d**	1	-CN	20
**66e**	1	-NO_2_	24
**66f**	2	-N(CH_3_)_2_	16
**66g**	2	-OMe	13
**66h**	2	-H	14
**66i**	2	-CN	25
**66j**	2	-NO_2_	20

### 3.6. Application of Suzuki-Miyaura Cross-Coupling in the Synthesis of Polysubstituted Quinazolinones and Quinazolines

**Scheme 21:**
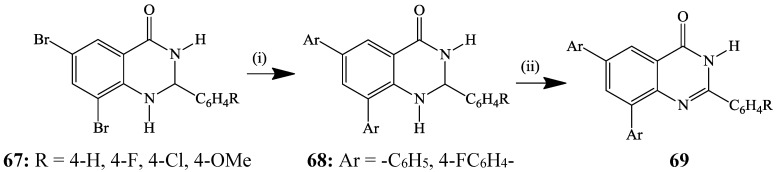
Suzuki-Miyaura cross-coupling of **67** and subsequent dehydrogenation of **68**.

Palladium-catalyzed Suzuki-Miyaura cross-coupling reaction between organoboron compounds and organic halides or triflates and tosylates is a general and efficient method for the formation of C*sp*^2^–C*sp*^2^ bonds [40]. This cross-coupling reaction has several advantages including functional group compatibility, low toxicity of reagents and intermediates, easy availability of boron derivatives, high thermal stability and good tolerance toward oxygen and aqueous solvents [40]. Suzuki-Miyaura cross-coupling reaction is an area of intense research for the synthesis of aryl- and arylvinyl-substituted quinazolinones and quinazolines with potential application in material and medicinal chemistry. Suzuki cross-coupling of the 2-aryl-6,8-dibromo-2,3-dihydroquinazolin-4(1*H*)-ones **67** with arylboronic acids yielded the corresponding 2,6,8-triaryl-2,3-dihydroquinazolin-4(1*H*)-ones **68** (77%–91%), which were in turn, dehydrogenated using iodine (2 equiv.) in ethanol under reflux to afford the potentially tautomeric 2,6,8-triarylquinazolin-4(3*H*)-ones **69** in 71%–96% yields (Scheme 21) [41]. A three layered greenish-yellow electroluminescent device based on the analogous 2-[4ꞌ-(N,N-dimethylaminophenyl)]-2,3-dihydroquinazolin-4(1*H*)-one as an emissive layer sandwiched between a hole transporting layer, *N,N′*-diphenyl-*N,N′*-bis(3-methylphenyl)-1,1-biphenyl-4,4'-diamine (TPD), and an electron transporting layer, tris(8-hydroxyquinolinato)aluminium (Alq3) {ITO/TPD/MAPQ/Alq3/Al} has been developed [42]. Likewise, quinazolin-4(3*H*)-one–based compounds exhibit interesting photophysical (electronic absorption and emission) properties [43]. The electronic absorption and emission spectra of 2-(4-methoxystyryl)-3-phenylquinazolin-4(3*H*)-one acquired in acetonitrile at 25 °C, for example, revealed that this compound absorbs in the ultra-violet region (λ_ab_ 348 nm) and emits in the visible region at λ_em_ 485 nm [44].

*Reagents and conditions*: (i) ArB(OH)_2_ (2.2 equiv.), PdCl_2_(PPh_3_)_2_, Xphos, K_2_CO_3_, DMF-water (4/1, v/v), 120 °C, 2 h; (ii) I_2_ (2 equiv.), ethanol, reflux, 2 h.

**Scheme 22:**
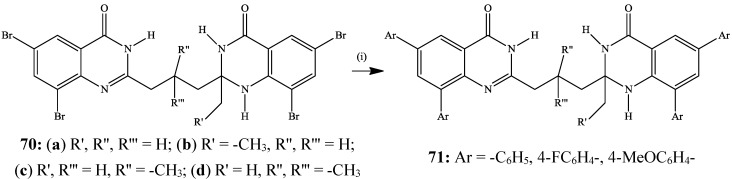
Suzuki-Miyaura cross-coupling of bis-heterocycles **70** with arylboronic acids.

The Suzuki-Miyaura cross-coupling of tetrabromobis-heterocycles **70** with arylboronic acids (4.5 equiv.) in the presence of PdCl_2_(PPh_3_)_2_-tricyclohexylphosphine (PCy_3_) catalyst complex in dioxane-water mixture (3/1, v/v) in the presence of K_2_CO_3_ under reflux afforded the corresponding tetraarylbis-heterocycles **71** in 60%–81% yield, exclusively (Scheme 22) [45]. Lack of selectivity in the cross-coupling is the consequence of equal C*sp*^2^–Br bond dissociation energies [15]. The electronic absorption and emission properties of these bis-heterocycles in dimethylsulfoxide (DMSO) and acetic acid in conjunction with quantum chemical methods showed a strong correlation with the substituents on the aryl groups and the size of the alkyl substituent on the 2-position of the quinazolin-4(1*H*)-one moiety as well as the substitution pattern on the propyl chain [45].

*Reagents and conditions*: (i) ArB(OH)_2_ (4.5 equiv.), PdCl_2_(PPh_3_)_2_, PCy_3_, K_2_CO_3_, dioxane–water (3/1, v/v), reflux, 5 h.

**Scheme 23:**
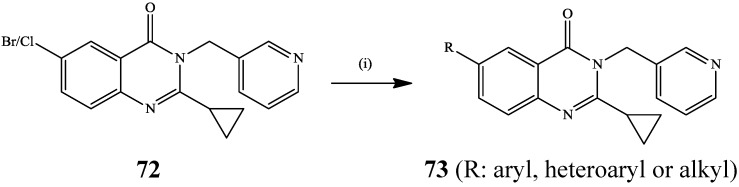
Suzuki cross-coupling of **72** with ArB(OH)_2_.

Previously, the Suzuki-Miyaura cross-coupling of furfurylboronic acid and 6-iodoquinazolin-4(3*H*)-one catalysed by Pd(OAc)_2_–tri-*t*-butylphosphine catalyst complex afforded 5-(4-oxo-3,4-dihydroquinazolin-6-yl)furan-2-carbaldehyde as a precursor for the synthesis of Lapatinib **3** [5]. Initial Suzuki-Miyaura cross-coupling of 2-(2-furyl)-6-iodoquinazolin-4(3*H*)-one with 4-methoxyphenylboronic in the presence of Pd(OAc)_2_-K_2_CO_3_ mixture in dioxane-water (3:1, v/v) and subsequent oxidative-aromatization into the 4-chloroquinazoline followed by amination has also been described in the literature [4]. The Suzuki-Miyaura cross-coupling of 6-bromo/chloro-2-cyclopropyl-3-((pyridin-3-yl)methyl)quinazolin-4(3*H*)-one **72** with aryl-, heteroaryl- and alkylboronic acids previously afforded 2-cyclopropyl-6-phenyl-3-((pyridin-3-yl)methyl)quinazolin-4(3*H*)-one **73** as potential α-glucosidase inhibitors (Scheme 23) [46]. Several palladium catalysts were used as sources of active Pd(0) species in the presence of K_3_PO_4_ in tetrahydropyran (THP) at room temperature and among them, bis-(dicyclohexylphosphino)ferrocene]dichloropalladium(II) [PdCl_2_(dcpf)] and bis(di-*tert*-butylphosphino) ferrocene]dichloropalladium(II) [PdCl_2_(dtbpf)] were found to be better catalysts that produced the desired products **73** in high yields. Although THP was found to be a superior solvent than dioxane at room temperature, it involved prolonged reaction time (36 h) and some of the arylboronic acids failed to react with the halogenated substrates. Complete transformation and improved yields were observed within 8 h when dioxane was used as solvent under reflux. Likewise, the use of 6-bromoquinazoline as substrate was found to be superior to 6-chloroquinazoline in terms of yields.

*Reagents and conditions*: (i) arylboronic acid, PdCl_2_(dcpf), K_3_PO_4_, dioxane, r.t.

**Scheme 24:**
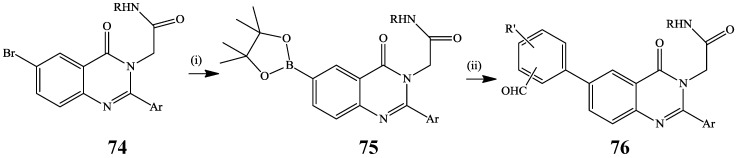
Suzuki-Miyaura cross-coupling of **74** with bis(pinacolato)diboron.

Napier *et al.* previously reacted the bromoquinazolinone derivative **74** with bis(pinacolato)diboron in the presence of PdCl_2_(dppf) to afford the boronate intermediate **75**, which upon Suzuki cross-coupling with bromobenzaldehyde derivatives afforded the 2,3,6-trisubstituted quinazolin-4(3*H*)-ones **76** (Scheme 24) [2]. The latter were, in turn, subjected to reductive amination to afford the corresponding 2-(6-aminomethylaryl-2-aryl-4-oxoquinazolin-(3(4*H*)-yl)acetamides with low nanomolar affinity for the vazsopressin V_1b_ and good selectivity with respect to related receptors V_1a_, V_2_ and OT [2].

*Reagents and conditions*: (i) bis(pinacolato)diboron, PdCl_2_(dppf)KOAc, DMF, 80 °C; (ii) bromobenzaldehydes, Pd(PPh_3_)_4_, K_3_PO_4_, DMF, 80 °C.

**Scheme 25:**
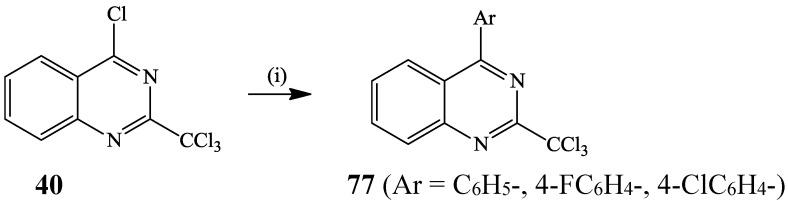
Suzuki-Miyaura cross-coupling of 4-chloro-2-trichloromethylquinazoline **40**.

4-(Dimethyl/phenylaminophenyl)quinazolines were recently prepared via the Suzuki-Miyaura cross-coupling of 4-chloroquinazoline with arylboronic acids and the compounds were found to exhibit absorption bands in the UV region and to emit green light upon irradiation [35]. Kieffer *et al.* also subjected 4-chloro-2-trichloromethylquinazoline **40** to Suzuki-Miyaura cross-coupling with arylboronic acids (2.5 equiv.) in the presence of Pd(OAc)_2_ in DMF to afford the corresponding 4-aryl-2-trichloromethylquinazolines **77** (50%–65%) with antiplasmodial properties (Scheme 25) [31].

*Reagents and Conditions*: (i) ArB(OH)_2_ (2.5 equiv), Pd(OAc)_2_, Cs_2_CO_3_, DMF, reflux, 2 h.

**Scheme 26:**
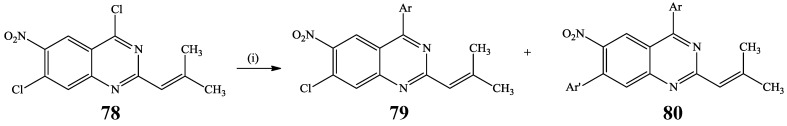
Suzuki-Miyaura cross-coupling of 4,7-dichloroquinazolines **78**.

The Suzuki-Miyaura cross-coupling of 4,7-dichloro-2-(2-methylprop-1-enyl)-6-nitroquinazoline **78** with arylboronic acids (1.5 equiv) in the presence of Na_2_CO_3_ and Pd(PPh_3_)_4_ in dimethoxyethane-ethanol mixture (9:1, v/v) under microwave irradiation afforded the C-4 substituted products **79** (Ar: 3-NO_2_Ph-, 5-Me-2-thienyl-, 4-ClPh-, 3-CF_3_Ph-) in 43%–78% yield, exclusively (Scheme 26) [47]. In the case of 4-methoxyphenyl derivative, the C-4 substituted product **79** (Ar = 4-MeOC_6_H_4_-) was only obtained in 68% yield using 1.2 equivalent of the 4-methoxyphenylboronic acid whereas the use of 1.5 equivalent afforded a mixture of **79** and the disubstituted product **80** in 45% and 15% yield, respectively. The disubstituted product **80** (Ar, Ar′ = 4-MeOC_6_H_4_-) on the other hand, was formed as the sole product in 70% yield when an excess of the 4-methoxyphenylboronic acid (4 equiv.) was used as a coupling partner. Under the same reaction conditions, **78** reacted with phenyl-, 4-fluorophenyl- and 2-tolylboronic acids to afford products **80** in 85, 71 and 65% yield, respectively (Scheme 26). Cross-coupling of **79** to afford the C-7 substituted product **80** was generally achieved using arylboronic acid (2 equiv.) in the presence of Pd(PPh_3_)_4_ and Na_2_CO_3_ in DMF/ethanol mixture (9:1, v/v) under microwave conditions for 3 h.

*Reagents and Conditions*: (i) ArB(OH)_2_, Na_2_CO_3_, Pd(PPh_3_)_4 _, DME/ethanol (9:1, v/v), MW (300 W), 80 °C, 3 h.

**Scheme 27:**
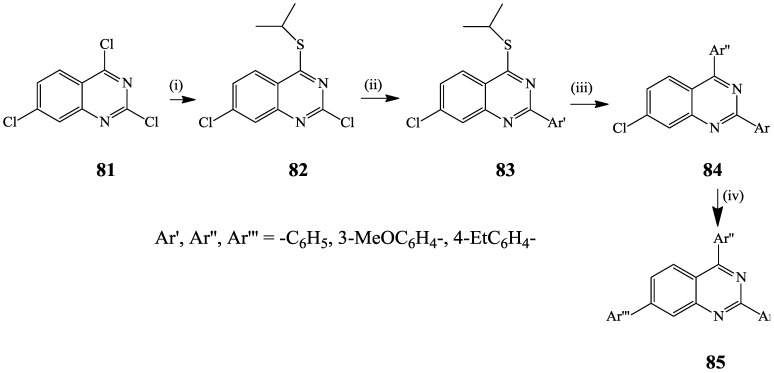
Sequential site-selective Suzuki-Miyaura cross-coupling of **81**.

Although Sonogashira cross-coupling of 2,4-dichloroquinazoline with terminal acetylenes occurs exclusively at the C-4 position, these substrates were found to undergo Pd(PPh_3_)_4_ catalyzed diarylation with arylboronic acids under reflux for 24 h to afford novel (*E,E*)-2,4-bis(dimethyl/ phenylaminophenyl)quinazolines with absorption bands in the UV region and they emit green light upon irradiation [35]. Hitherto, regioselective Pd-catalyzed Suzuki-Miyaura cross-coupling reaction of 2,4,7-trichloroquinazoline **81** with aryl- and heterarylboronic acids was reported to favor coupling at C-4 position albeit in low yield due to competitive hydrolysis at this site [48]. The authors, in turn, temporarily deactivated the C-4 position as thioether **82** to effect regioselective Suzuki-Miyaura cross-coupling with arylboronic acid derivatives at the C-2 position to yield **83** (Scheme 27). A second Suzuki-Miyaura cross-coupling with arylboronic acids using Pd(PPh_3_)_4_–copper(I) thiophene-2-carboxylate (CuTC) catalyst complex in THF under reflux afforded the C-4 arylated product **84**, which was in turn arylated at the C-7 position to afford trisubstituted quinazolines **85** [48].

*Reagents and conditions*: (i) propane-2-thiol, NaH, degassed THF, r.t.; (ii) Ar'B(OH)_2_, Pd(OAc)_2_, PPh_3_, Na_2_CO_3_, DME and H_2_O (10:1, v/v), 75 °C, N_2_; (iii) Ar''B(OH)_2_, Pd(PPh_3_)_4_, CuTC, THF, 50 °C, N_2_ atmosphere; (iv) Ar'''B(OH)_2_, Pd(OAc)_2_, PPh_3_, Na_2_CO_3_, DME:H_2_O (10:1, v/v), reflux.

**Scheme 28:**
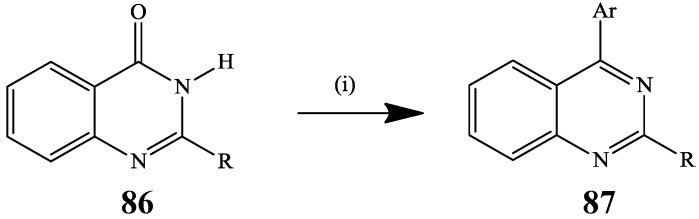
Suzuki-Miyaura cross-coupling of tosylates generated *in situ* from **86**.

Amination of 4-chloro-2-(furan-2-yl)-6-iodoquinazoline with isopropylamine in propan-2-ol followed by Suzuki-Miyaura cross-coupling of 2-(furan-2-yl)-6-iodo-*N*-isopropylquinazoline with arylboronic acids in the presence of palladium acetate and potassium carbonate in aqueous dioxane under reflux previously afforded a series of 4-amino-6-arylquinazoline [4]. An *in situ* C–OH activation of the 2-substituted quinazolin-4(3*H*)-ones **86** as tosylates followed by Suzuki-Miyaura cross-coupling with arylboronic acids previously afforded a series of 2,4-diarylquinazolines **87** in a single-pot operation (Scheme 28) [49].

**Scheme 29:**
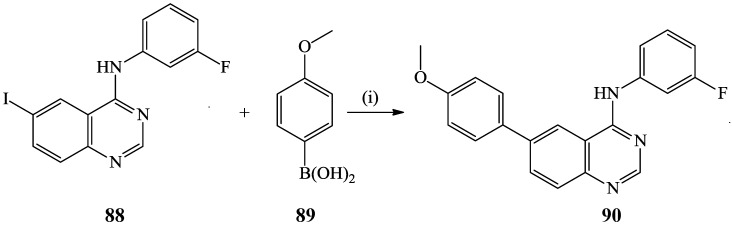
The Suzuki cross-coupling reaction of *N*-(3-fluorophenyl)-6-iodoquinazolin-4-amine.

The Suzuki-Miyaura cross-coupling of *N*-(3-fluorophenyl)-6-iodoquinazolin-4-amine **88** with 4-methoxyphenylboronic acid **89** using Pd(OAc)_2_ as a catalyst in the presence of Cs_2_CO_3_ in aqueous dioxane afforded the *N-(*3-fluorophenyl)-6-(4-methoxyphenyl)quinazolin-4-amine **90** in 70% yield (Scheme 29) [4]. Cross-coupling of the analogous 6-iodo-*N*-phenylquinazolin-4-amine with arylboronic acids in the presence of PdCl_2_(PPh_3_)_2_-PPh_3_ catalyst complex, Cs_2_CO_3_ as a base in DMF under reflux for 24 h afforded the corresponding 6-aryl-*N*-phenylquinazolin-4-amines albeit in low yields [39]. These compounds were found to exhibit ERBB2-induced fluorescence and to represent a turn-on probe that can report binding to the kinase domain in organic solutions [39].

*Reagents and Conditions*: (i) ArB(OH)_2_, K_2_CO_3_, PdCl_2_(PPh_3_)_2_, TsCl, THF–H_2_O (20:1, v/v), 60 °C.

**Table:** 

	R	Ar	%Yield
**87a**	C_6_H_5_	-C_6_H_5_	85
**87b**	-C_6_H_5_	4-MeC_6_H_4_-	88
**87c**	-C_6_H_5_	4-MeOC_6_H_4_-	90
**87d**	-C_6_H_5_	4-ClC_6_H_4_-	79
**87e**	4-MeC_6_H_4_-	-C_6_H_5_	83
**87f**	4-MeC_6_H_4_-	4-MeC_6_H_4_-	85
**87g**	4-MeC_6_H_4_-	4-MeOC_6_H_4_-	85
**87h**	4-MeC_6_H_4_-	4-ClC_6_H_4_-	79
**87i**	4-MeOC_6_H_4_-	-C_6_H_5_	81
**87j**	4-MeOC_6_H_4_-	4-ClC_6_H_4_-	75
**87k**	4-MeOC_6_H_4_-	4-MeC_6_H_4_-	83
**87l**	4-ClC_6_H_4_-	-C_6_H_5_	85
**87m**	4-ClC_6_H_4_-	4-ClC_6_H_4_-	80
**87n**	4-ClC_6_H_4_-	4-MeC_6_H_4_-	90
**87o**	4-ClC_6_H_4_-	4-MeOC_6_H_4_-	75
**87p**	-CH_2_CH_2_CH_3_	-C_6_H_5_	63
**87q**	-CH(CH_3_)_2_	-C_6_H_5_	43

*Reagents and Conditions*: (i) Cs_2_CO_3_, Pd(OAc)_2_, dioxane–water (3:1, v/v), reflux, 30 min.

**Scheme 30:**
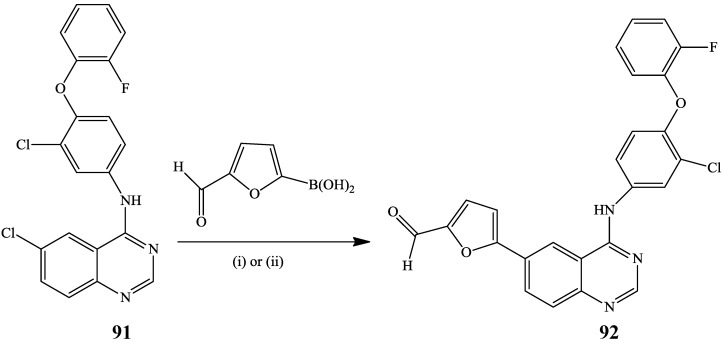
Pd/C catalysed Suzuki-Miyaura cross-coupling of **91**.

Initial Suzuki-Miyaura cross-coupling of 4-amino-6-iodoquinazoline **91** and furfurylboronic acid in the presence of 10% Pd/C and triethylamine in ethanol under reflux (i) or through C–H activation using PdCl_2_-KOAc mixture (ii) previously afforded the 4-anilino-6-furfurylquinazoline **92**, a precursor for the synthesis of Lapatinib **3** (Scheme 30) [5].

*Reagents and conditions*: (i) 10% Pd/C, NEt_3_, EtOH, 60 °C; (ii) PdCl_2_, KOAc.

**Scheme 31:**
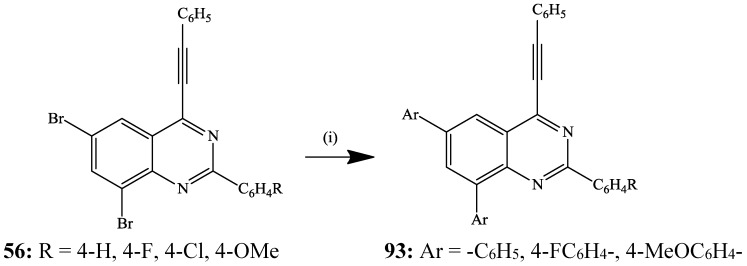
Suzuki-Miyaura cross-coupling of **56** with arylvinylboronic acid.

A series of 2-aryl-6,8-dibromo-4-(arylethynyl)quinazolines **56** was, in turn, subjected to the Suzuki-Miyaura cross-coupling with arylboronic acids in the presence of PdCl_2_(PCy_3_)_2_ and K_2_CO_3_ in dioxane under reflux for 4 h to afford the corresponding 2,6,8-triaryl-4-(2-phenylethynyl)quinazolines **93** with potential photophysical properties (Scheme 31) [37]. Lack of selectivity for the cross-coupling was attributed to comparable C*sp*^2^–Br bond strengths based on the literature precedents on the analogous 2-aryl-6,8-dibromo-4-methoxyquinolines [50] and tribromoquinoline [51].

*Reagents and conditions:* (i) ArB(OH)_2_, PdCl_2_(PCy_3_)_2_, K_2_CO_3_, dioxane, reflux, 4 h.

**Scheme 32:**
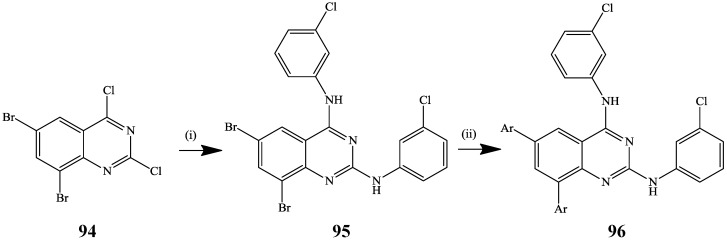
Single-pot amination and Suzuki-Miyaura cross-coupling of **94**.

Microwave-assisted consecutive bis-S_N_Ar/bis-Suzuki cross-coupling reaction involving initial amination of 6,8-dibromo-2,4-dichloroquinazoline **94** afforded 6,8-dibromo-*N*^2^,*N*^4^-bis(3-chlorophenyl) quinazoline-2,4-diamine **95**, which upon cross-coupling with arylboronic acids (2 equiv.) yielded the corresponding 2,4,6,8-tetrasubstituted quinazolines **96** without selectivity (Scheme 32) [52]. Moreover, the authors performed these consecutive steps in a single-pot operation to afford products **96**, exclusively.

*Reagents and conditions*: (i) 3-ClC_6_H_4_NH_2_, DME,-EtOH (9:1, v/v), MW, 100 °C; (ii) ArB(OH)_2_, PdCl_2_(PPh_3_)_2_, K_2_CO_3_, H_2_O, MW, 100 °C, 1 h.

### 3.7. Application of Buchwald-Hartwig Cross-Coupling in the Synthesis of Polyheteroatom-Substituted Quinazolinones

**Scheme 33:**
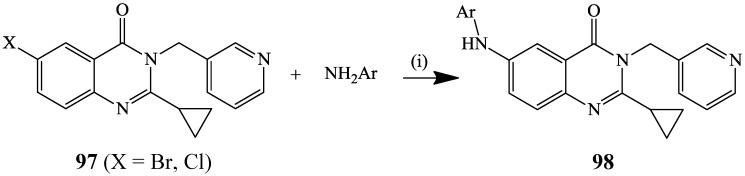
Palladium catalyzed amination of **97**.

The Buchwald-Hartwig involves the direct C–N or C–O bond formation between aryl- or heteroaryl halides and amines or alcohols in the presence of stoichiometric amount of a base and palladium as a catalyst. This reaction was previously applied on 6-bromo- and 6-chloro-2-cyclopropyl-3-(pyridyl-3-methyl)quinazolin-4(3*H*)-ones **97** using a variety of aryl, heteraryl and alkyl amines as coupling partners in the presence of Pd_2_(dba)_3_–DavePhos catalyst complex and *t-*BuONa in 1,4-dioxane at 100 °C to afford the corresponding 6-aminated derivatives **98** in moderate to high yields (Scheme 33) [53].

*Reagents and conditions*: (i) Pd_2_(dba)_3_, DavePhos, *t*-BuONa, dioxane, 100 °C, 8 h.

**Scheme 34:**
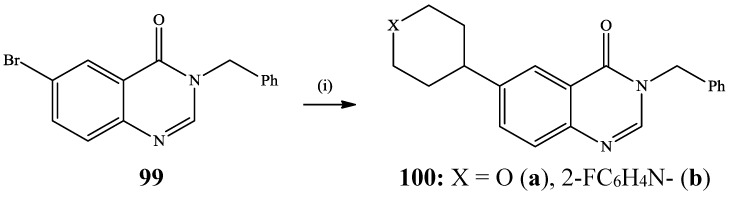
Buchwald-Hartwig amination of quinazolin-4(3*H*)-one **102**.

Buchwald-Hartwig cross-coupling of 6-bromoquinazolin-4(3*H*)-one **99** with morpholine or 1-(2-fluorophenyl)piperizine in the presence palladium acetate-xantphos catalyst complex and potassium *tert-*butoxide in dioxane under reflux recently afforded the 6-(morpholin-4-yl)quinazolin-4(3*H*)-ones **100a** (X = O) and **100b** (X = 2-FC_6_H_4_N**-**) in 89% and 88% yield, respectively (Scheme 34) [54]. Under the same reaction conditions, the 3-(4-methoxybenzyl)- and 3-benzyl-6-bromobenzo[*h*]quinazolin-4(3*H*)-one derivatives afforded the corresponding 6-(morpholin-4-yl)benzo[*h*]quinazolin-4(3*H*)-one derivatives in 45% and 40% yield, respectively. The 3-(4-methoxybenzyl)-6-(morpholin-4-yl)benzo[*h*]quinazolin-4(3*H*)-one was found to be highly toxic against HT29 cancer cells (IC_50_ = 4.12 μM) [54].

*Reagents and conditions*: (i) morpholine or 1-(2-fluorophenyl)piperizine, Pd(OAc)_2_, *t-*BuOK, toluene, 100 °C, 20 h.

**Scheme 35:**
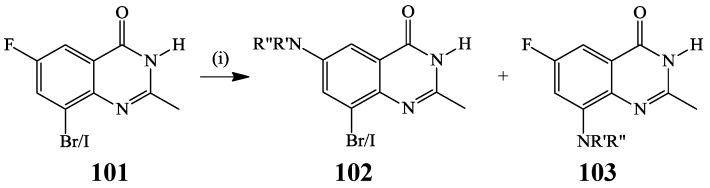
Regioselective C-6 amination of **101**.

Baarlaam *et al.* performed the amination of 3-(*p*-methoxybenzyl) protected 8-bromo-6-fluoro-2-methylquinazolino-4(3*H*)-one **101** with primary and secondary amines without the use of transition metal catalyst and isolated 6-amino substituted products **102** as major or sole products (Scheme 35) [55]. A halogen atom (X = Br, I) *meta* to the fluorine leaving group is envisioned to exert a sufficient inductive effect to permit the substitution under standard laboratory conditions. However, this reaction could not be extended to the alkoxides due to deprotonation and deactivation of the quinazoline ring towards substitution [55].

*Reagents and conditions*: (i) R'R''NH, 140 °C, 3 days.

**Table:** 

	R', R''	%Yield 102	%Yield 103
**a**	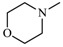	68	12
**b**	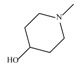	79	9
**c**	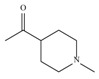	68	7
**d**	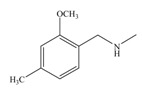	45	-
**e**	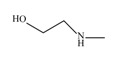	41	-
**f**	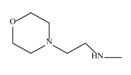	43	-
**g**	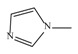	39	-

### 3.8. Palladium Catalyzed Cyanation in the Synthesis of Polysubstituted Quinazolines

**Scheme 36:**
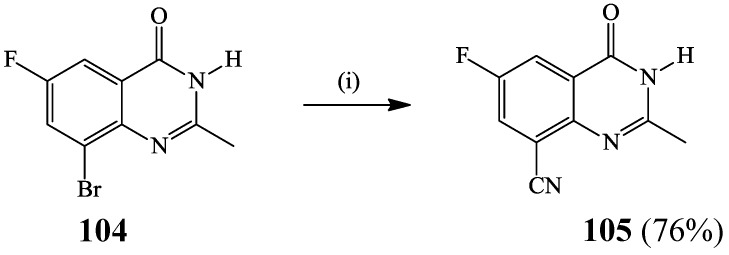
Palladium catalyzed C-8 cyanation of **104** with zinc cyanide.

Cyanation of aromatic halides in the presence of palladium(0) catalyst source has proven to be superior to the Rosemund-Von Braun reaction which employs stoichiometric amount of CuCN under high temperature conditions accompanied by tedious work-up [56]. An excess of the strong σ-donor cyanide ion which has high affinity for palladium tend to poison the catalyst and, in turn, retard the progress of reaction [56]. A microwave assisted cyanation of 8-bromo-6-fluoro-2-methylquinazolino-4(3*H*)-one **104** using zinc cyanide in the presence of tris(dibenzylideneacetone)dipalladium(0) (Pd_2_(dba)_3_)–xanthphos catalyst complex afforded the C8–CN substituted product **105**, exclusively (Scheme 36) [55]. Zn(CN)_2_ in this case serves as a source of low concentration of cyanide ion during the reaction to avoid poisoning the catalyst.

*Reagents and conditions*: (i) Zn(CN)_2_, Pd_2_(dba)_3_, xanthphos, DMA, 160 °C MW, 10 min.

**Scheme 37:**
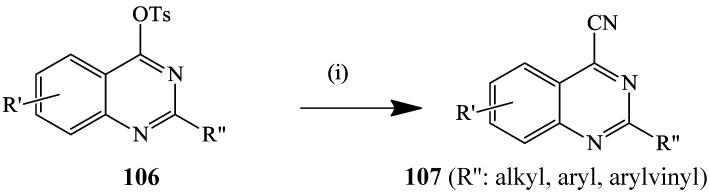
Palladium catalyzed cyanation of 4-tosylquinazolines with CuCN.

A series of 2-substituted 4-tosylquinazolines **106** was recently subjected to copper(I) cyanide (2 equiv.) in the presence of palladium(II) acetate–bis(diphenylphosphino)ferrocene (DPPF) catalyst complex and cesium carbonate in toluene under reflux to yield the corresponding 4-cyanoquinazoline **107** in moderate to high yields (Scheme 37) [57]. Under the same conditions, 4-chloro-2-(4-methoxyphenyl)quinazoline was found to be less reactive and to afford the corresponding 4-cyanoquinazoline in 11% yield. 

*Reagents and conditions*: (i) CuCN, Pd(OAc)_2_, DPPF, Cs_2_CO_3_, toluene, 80 °C, 8–12 h.

The nitrile group offers a useful functionality for subsequent manipulations to important functional groups such as hydrolysis into acids, hydration into amides, reduction into amines or aldehydes, and cycloaddition into various heterocycles.

## 4. Conclusions and Perspective

The observed reactivity of the dihalogenated quinazolinones in metal catalyzed C-C bond formation follows a similar trend to that observed for the other aryl/heteroaryl halides (C-I > C-Br >> C-Cl), whereby selective coupling occurs with the more intrinsically reactive iodides or bromides in the presence of chlorides. The C(4)-Cl bond of quinazoline derivatives, on the other hand, is highly activated than the other positions bearing Cl or Br due to α-nitrogen effect and in general, the oxidative addition of Pd(0) to a C(4)-Cl bond occurs easily at room temperature without the use of specialized and expensive ligands. Moreover, the 4-chloroquinazoline moiety is preferred over the 4-bromo- or 4-iodoquinazoline framework in the cross-coupling reactions [31]. Despite the successes in site-selective or sequential metal-catalyzed halogen substitution reactions, the application of this strategy towards the synthesis of polysubstituted quinazolinones and quinazolines from the corresponding di- or trihalogenated precursors in a single-pot operation remains a challenge. In our view, this could be realized through the use of di- or trihalogenated quinazolinones or quinazolines bearing mixed halogen atoms to increase the diversity of substitution on the heterocyclic scaffold/s to afford compounds with interesting biological or photophysical properties. Heterocyclic compounds with intramolecular charge transfer properties continue to attract considerable attention for potential applications in organic electroluminescent diodes, organic solar cells, polarity probes and nonlinear optics. Polycarbo-substituted quinazolinones and quinazoline derivatives comprise electron-deficient heterocyclic scaffolds (quinazolinone or quinazoline framework) as electron-acceptors linked directly to the aryl ring or through π-conjugated bridge/s to comprise donor-π-acceptor systems with intramolecular charge transfer properties. Given the need for efficient methods for the incorporation of quinazolinone and quinazoline moieties in pharmaceutical compounds or materials, it can be expected that growth and exciting new advances will continue in this important subdomain of metal-catalyzed cross-coupling research.
